# Decrease in Urinary Creatinine Excretion in Early Stage Chronic Kidney Disease

**DOI:** 10.1371/journal.pone.0111949

**Published:** 2014-11-17

**Authors:** Elena Tynkevich, Martin Flamant, Jean-Philippe Haymann, Marie Metzger, Eric Thervet, Jean-Jacques Boffa, François Vrtovsnik, Pascal Houillier, Marc Froissart, Bénédicte Stengel, on behalf of the NephroTest Study Group

**Affiliations:** 1 CESP, Centre for Epidemiology and Population Health, INSERM Unit 1018, Villejuif, France; 2 University Paris-Sud 11, UMRS 1018, Villejuif, France; 3 AP-HP, Hôpital Bichat, Department of Physiology, Paris, France; 4 AP-HP, Hôpital Tenon, Department of Physiology, Paris, France; 5 INSERM UNIT 702, Paris, France; 6 University Pierre et Marie Curie-Paris 6, UMRS 702, Paris, France; 7 AP-HP, Hôpital Européen Georges Pompidou, Department of Nephrology, Paris, France; 8 AP-HP, Hôpital Européen Georges Pompidou, DHU Common and Rare Arterial Diseases, Paris, France; 9 AP-HP, Hôpital Tenon, Department of Nephrology, Paris, France; 10 AP-HP, Hôpital Bichat, Department of Nephrology, Paris, France; 11 University Paris Descartes-Paris 5, UMRS 775, Paris, France; 12 AP-HP, Hôpital Européen Georges Pompidou, Department of Physiology, Paris, France; University Medical Center Utrecht, Netherlands

## Abstract

**Background:**

Little is known about muscle mass loss in early stage chronic kidney disease (CKD). We used 24-hour urinary creatinine excretion rate to assess determinants of muscle mass and its evolution with kidney function decline. We also described the range of urinary creatinine concentration in this population.

**Methods:**

We included 1072 men and 537 women with non-dialysis CKD stages 1 to 5, all of them with repeated measurements of glomerular filtration rate (mGFR) by ^51^Cr-EDTA renal clearance and several nutritional markers. In those with stage 1 to 4 at baseline, we used a mixed model to study factors associated with urinary creatinine excretion rate and its change over time.

**Results:**

Baseline mean urinary creatinine excretion decreased from 15.3±3.1 to 12.1±3.3 mmol/24 h (0.20±0.03 to 0.15±0.04 mmol/kg/24 h) in men, with mGFR falling from ≥60 to <15 mL/min/1.73 m^2^, and from 9.6±1.9 to 7.6±2.5 (0.16±0.03 to 0.12±0.03) in women. In addition to mGFR, an older age, diabetes, and lower levels of body mass index, proteinuria, and protein intake assessed by urinary urea were associated with lower mean urinary creatinine excretion at baseline. Mean annual decline in mGFR was 1.53±0.12 mL/min/1.73 m^2^ per year and that of urinary creatinine excretion rate, 0.28±0.02 mmol/24 h per year. Patients with fast annual decline in mGFR of 5 mL/min/1.73 m^2^ had a decrease in urinary creatinine excretion more than twice as big as in those with stable mGFR, independent of changes in urinary urea as well as of other determinants of low muscle mass.

**Conclusions:**

Decrease in 24-hour urinary creatinine excretion rate may appear early in CKD patients, and is greater the more mGFR declines independent of lowering protein intake assessed by 24-hour urinary urea. Normalizing urine analytes for creatininuria may overestimate their concentration in patients with reduced kidney function and low muscle mass.

## Introduction

Protein-energy wasting (PEW) is a state of decreased body stores of protein and energy fuels, assessed by biochemical and clinical measures [1]–[4]. Nutritional disorders are common in patients with end-stage chronic kidney disease (CKD) [1], [5]–[8] and are often associated with inflammation [7], [9]–[12]. Both conditions are known to be associated with high cardiovascular [10], [11], [13] and all-cause [5], [10], [14]–[17] mortality. While nutritional disorders have previously been studied in patients with end-stage CKD [1], [6], [14], [16], [18]–[24] or coronary heart disease [13], [25], and in the general population [26]–[29], little is known about the timing of PEW in non-end-stage CKD [17], [30].

Muscle wasting is one of the most valid markers of PEW [2], [8], [14]. Loss of muscle mass results from an imbalance between protein synthesis and degradation and is worsened by inactivity [22], [31] and loss of appetite [9], [32], [33]. A variety of conditions, including diabetes mellitus [21], [24], metabolic acidosis [34], [35], and inflammation [19], [31], [36], [37], may promote an increase in protein breakdown. Muscle mass may be assessed by biochemical [2], [3], [38], [39], anthropometric [2], [3], or bioelectrical measurements [40], [41]. In steady state, urinary creatinine excretion is one of the most specific indexes of total body muscle mass, because creatine originates almost exclusively (98%) from skeletal muscle[39], [42]. The amount of both filtered and secreted creatinine excreted in the urine represents the difference between creatinine generation and its extrarenal elimination. The former is proportional to muscle mass but also affected by meat intake [3], [39], [42], and the latter is known to increase in patients with severely reduced kidney function [42], [43]. Several studies reported the normal range and determinants of urinary creatinine concentration in the general population[27]. Very few, however, have investigated its relation to kidney function decline, while taking into account the determinants of muscle mass, including reduced protein intake [17], [30]. Moreover, most studies which investigated the association between urinary excretion of creatinine and renal function were based on glomerular filtration rate (GFR) estimated from plasma creatinine concentration, which is itself determined by muscular production of creatinine (and hence its urinary excretion). Thus, using a gold standard GFR measurement method, which is independent of plasma creatinine concentration, is methodologically crucial to study the correlation between urinary creatinine excretion and renal function decline.

We therefore studied the determinants of low urinary creatinine excretion as well as of the evolution of its rate over time, in a large cohort of patients with all stages of CKD and measured glomerular filtration rate (mGFR) from the NephroTest study. In addition, we document the range of urinary creatinine concentrations by age, gender, ethnicity and mGFR level.

## Methods

### Ethics statement

All patients signed written informed consents before inclusion in the cohort. The NephroTest study design was approved by an ethics committee (Direction générale pour la recherche et l'innovation. Comité consultatif sur le traitement de l'information en matière de recherche dans le domaine de la santé (CCTIRS). Ref: DGRI CCTIRS MG/CP09.503, 9th July 2009).

### Patients and study design

The NephroTest study is a prospective hospital-based cohort that began in 2000; by the end of 2010, it had enrolled 1827 adult patients with all stages of CKD and all nephropathy types referred by nephrologists to any of three departments of physiology for annual extensive workups. Eligible patients were ≥18 years of age at inclusion and had neither started dialysis nor received a kidney transplant. Pregnant women were excluded.

### Information

Data were recorded during a 5-hour in-person visit for a complete nephrological workup comprising a large set of clinical and laboratory measurements, including blood pressure, body mass index (BMI), and treatments received. Diabetes was defined as either fasting glycemia ≥7 mmol/L or HbA1c ≥6.5% or antidiabetic treatment, hypertension by either a systolic or diastolic blood pressure >140/90 mm Hg or antihypertensive treatment, metabolic acidosis as venous CO_2_ <22 mmol/L or alkaline treatment.

### Laboratory measurements

**Figure 1:**
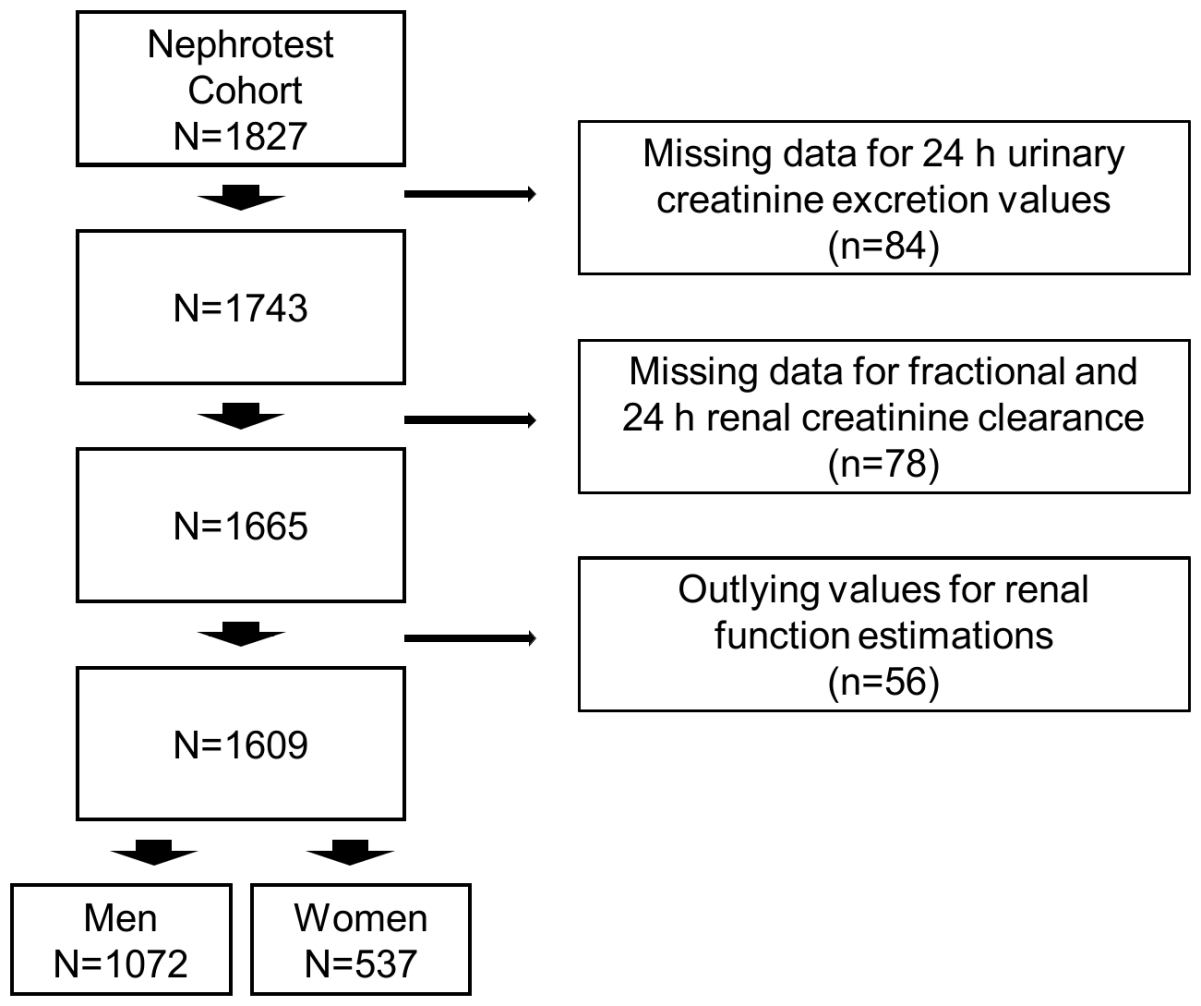
Flowchart of study sample.

At each visit, mGFR was measured concurrently for all patients by ^51^Cr-EDTA renal clearance and fractional creatinine clearance. Subjects also provided 24-hour urine collection, which enabled us to measure 24-hour creatinine clearance as well as creatininuria with the Jaffe method. Because 24-hour urine collection may be inaccurate, we primarily used 24-hour urinary creatinine extrapolated from fractional creatinine clearance in this study, on the assumption that creatinine excretion is stable over the 24-hour period. Moreover, the analysis of consistency between the two measures of creatinine clearance and that of mGFR enabled us to exclude outliers and to assess the completeness of the 24-hour urine collection. Thus, in addition to 84 patients with missing urinary creatinine, we excluded 78 patients with missing data for fractional creatinine clearance and 56 outliers for mGFR or for fractional or 24-hour creatinine clearance or both, leaving 1609 patients for the analysis of baseline data (Figure 1).

Gender-specific thresholds for low urinary creatinine excretion were defined as values <10^th^ percentile of the distribution in patients with mGFR ≥60 mL/min/1.73 m^2^, i.e., 11.4 mmol/24 h in men and 7.2 in women. Several markers of inflammation-malnutrition were also measured and studied, with the following cutoffs to define abnormal values: serum albumin <35 g/L, prealbumin <0.30 g/L, total cholesterol <5.5 mmol/L, triglycerides ≥1.7 mmol/L, transferrin <2 g/L, white blood cells (WBC) ≥7.5×10^3^/mm^3^, C-reactive protein (CRP) ≥10 mg/L, haptoglobin ≥90^th^ percentile, orosomucoid ≥90^th^ percentile, and plasma fibrinogen ≥5 g/L. Gender-specific thresholds were used to define high-density lipoprotein (HDL) cholesterol cutoff points: 1.0 mmol/L in men and 1.3 mmol/L in women. Urinary urea (mmol/24-hour), reflecting protein intake, and 24-hour proteinuria, were analyzed in quartiles.

Missing values accounted for less than 5% of most indicators. Mean imputation was used for those included in the multivariate analysis. A missing category was created for the indicators with >5% of values missing, including prealbumin (24%), orosomucoid (37%), and haptoglobin (21%).

### Statistical analyses

Variables were expressed as percentages, means ± SD, or medians (IQR: interquartile range), as appropriate. We first described patients' characteristics according to mGFR classes defined as follows: ≥ 0, 45-59, 30–44, 15–29, and <15 mL/min/1.73 m^2^. Differences across mGFR classes were tested with ANOVA for continuous variables and with Chi-square test for categorical variables. We also provided urinary creatinine mean values and 10^th^, 25^th^, 75^th^, and 90^th^ percentiles expressed in both mmol/24-h and mmol/kg/24-h, according to mGFR classes, overall and by age (< or ≥60 years), gender, and ethnicity (non-African *vs* African origin).

Crude and adjusted odds ratios (ORs) and 95% confidence intervals (95% CI) for low baseline urinary creatinine excretion according to clinical and laboratory parameters were estimated by logistic regression. To be included in the multivariate analysis, factors had to be significantly associated with low urinary creatinine excretion in the crude analysis with p<0.20 and uncorrelated with each other (−0.50<r<0.50). Well-established risk factors for muscle mass loss, such as diabetes, were also included regardless of their statistical significance in the crude analysis. We studied three models: one crude, one adjusted only for age (treated continuously), gender, ethnicity, center, and mGFR, and another fully adjusted. These analyses were also carried out by replacing mGFR by estimated GFR (eGFR) using the CKD-EPI equation[44], and by sub-group defined by diabetes status.

Finally, we used a linear mixed model with random intercepts and slopes to estimate mean differences in urinary creatinine excretion rate according to covariates shown to be statistically significant in the logistic regression, as well as its annual slope associated with changes in mGFR and 24-hour urinary urea. In this analysis, we excluded 99 patients with CKD stage 5 at baseline and 37 with missing urinary urea, leaving 1473 patients with a median follow-up of 1.43 years (0–3.34, maximum 5 years). Median interval between visits was 1.12 year (1.00–1.69) for the 855 patients with at least two visits. This analysis was based on a total of 3268 observations. Interactions with time were tested for all covariates. In the final mixed model, we only included interaction terms which were statistically significant using Wald test and improved the model according to Akaike information criteria (AIC).

A two-sided P-value <0.05 indicated statistical significance. Statistical analyses were performed with SAS software, version 9.3 (SAS institute, Cary, NC).

## Results

### Patient characteristics according to mGFR level

Patients' age increased and the percentage of those from sub-Saharan Africa or the French West Indies decreased with decreasing mGFR (Table 1). The level of all nutritional factors, except prealbumin, significantly decreased with mGFR decline, while that of inflammation factors (except CRP) as well as the prevalence of metabolic acidosis significantly increased.

**Table 1:** Patient characteristics according to mGFR level in 1609 patients.

	mGFR (ml/min per1.73 m^2^)					p Value
	≥60	45–60	30–44	15–29	<15	
	(n = 282)	(n = 321)	(n = 472)	(n = 435)	(n = 99)	
**Demographic parameters**						
Men	188 (66.7)	233 (72.6)	321 (68.0)	274 (63.0)	56 (56.6)	0.01
Age (years)	52.0±15.1	58.7±14.4	61.0±15.0	61.0±15.0	60.9±15.2	<0.0001
African origin	53 (18.8)	48 (15.0)	56 (11.9)	44 (10.1)	8 (8.1)	<0.0001
**Body mass index (kg/m^2^)**	25.9±4.9	26.3±4.9	26.6±4.8	26.6±5.0	27.0±6.0	0.19
Diabetes^a^	62 (22.0)	83 (25.9)	150 (31.8)	136 (31.3)	25 (25.3)	0.02
Metabolic acidosis^b^	6 (2.1)	11 (3.4)	47 (10.0)	100 (23.0)	46 (46.5)	<0.0001
Hypertension^c^	225 (79.8)	284 (88.5)	447 (94.7)	415 (95.4)	98 (99.0)	<0.0001
Any antihypertensive treatment	216 (76.6)	272 (84.7)	435 (92.2)	408 (93.8)	96 (97.0)	<0.0001
**Laboratory parameters**						
eGFR	75.9±19.6	54.3±12.1	39.5±9.8	25.4±8.5	13.7±5.6	<0.0001
HDL cholesterol (mmol/L)	1.36±0.40	1.35±0.42	1.30±0.44	1.24±0.39	1.29±0.50	0.001
Triglycerides (mmol/L)	1.1 (0.82–1.6)	1.2 (0.87–1.6)	1.4 (0.94–2.0)	1.4 (1.0–2.1)	1.5 (1.1–2.3)	<0.0001
Serum albumin (g/L)	40.2±3.7	40.3±3.9	39.3±4.5	38.8±4.6	38.9±5.0	<0.0001
Prealbumin (g/L)	0.29±0.07	0.30±0.06	0.30±0.07	0.33±0.0.8	0.34±0.08	<0.0001
Transferrin (g/L)	2.4±0.42	2.3±0.37	2.3±0.41	2.2±0.40	2.13±0.36	<0.0001
Homocysteine (umol/L)	13.5±4.3	16.9±6.1	19.1±6.3	21.7±7.6	24.0±7.8	<0.0001
Urinary urea (mmol/24h)	405.6±118.9	381.2±110.7	373.3±124.7	354.4±113.7	297.4±100.8	<0.0001
WBC count (10^3^/mm^3^)	6.1±1.8	6.2±2.0	6.5±2.3	6.8±2.2	6.7±1.9	0.0001
Plasma fibrinogen (g/L)	3.4±0.81	3.5±0.88	3.8±0.93	4.2±1.00	4.4±0.99	<0.0001
CRP*>10 *mg/L	15 (5.3)	23 (7.2)	43 (9.1)	50 (11.5)	8 (8.1)	0.20
Orosomucoid (g/L)	0.79±0.21	0.85±0.22	0.92±0.25	1.0±0.31	1.1±0.23	<0.0001
Haptoglobin (g/L)	1.2±0.58	1.2±0.58	1.4±0.61	1.5±0.67	1.5±0.69	<0.0001
Proteinuria (g/24h)	0.16 (0.10–0.50)	0.17 (0.11–0.40)	0.26 (0.13–0.93)	0.65 (0.23–1.8)	1.3 (0.55–2.3)	<0.0001

Abbreviations: BP, blood pressure; mGFR, measured glomerular filtration rate; HDL, high-density lipoprotein; WBC, white blood cells; CRP, C-reactive protein.

Values are reported as %, mean ± SD or median (interquartile range).

a Fasting glucose ≥7 mmol/L or HbA1c ≥6.5 or antidiabetic treatment or reported diabetes.

b Venous CO2 <22 mmol/L or alkaline treatment.

c Any antihypertensive treatment or systolic BP>140 or diastolic BP>90 mm Hg.

### Urinary creatinine excretion rate according to GFR level, by gender, age, and ethnicity

**Figure 2:**
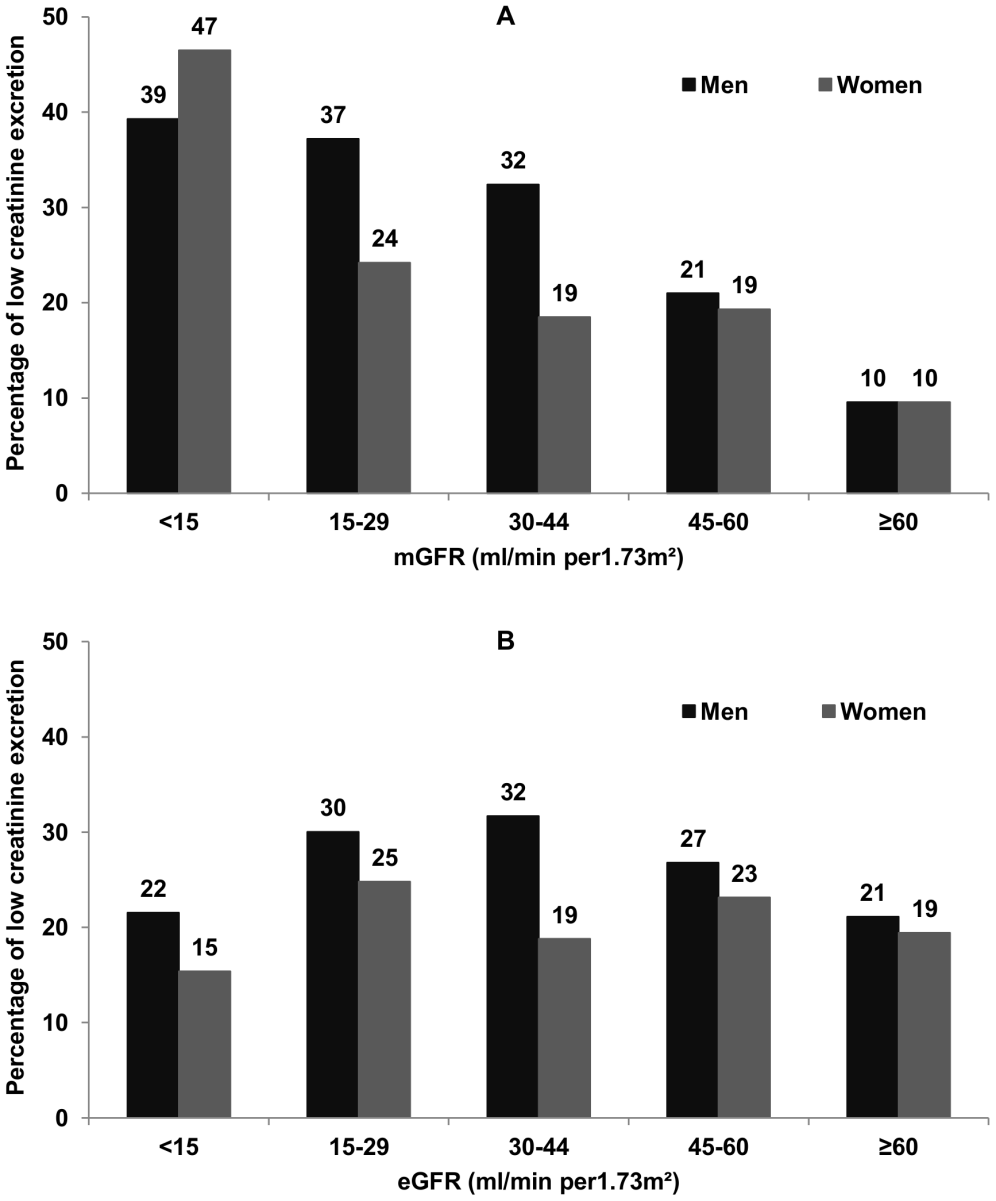
Percentages of low urinary creatinine excretion according to mGFR and eGFR level, by gender. Figure shows the percentage of patients with low creatinine excretion rates according to both measured (2A) and estimated (2B) glomerular filtration rate classes in men and women. Gender-specific thresholds were defined as the 10^th^ percentile of the urinary creatinine distribution in patients with mGFR ≥60 mL/min/1.73 m^2^.

Urinary creatinine excretion was normally distributed. Mean urinary creatinine excretion rate decreased as mGFR decreased from ≥60 to <15 mL/min/1.73 m^2^ in all categories defined by age, gender, and ethnicity (Table 2). It was higher in men and women with than without African origin. The percentage of patients with low creatinine excretion rates increased as mGFR decreased, reaching about four times the level of the reference category in those with mGFR less than 15 mL/min/1.73 m^2^ in both genders (Figure 2A). Of note, this percentage was twice as high in patients with GFR ≥ 0 mL/min/1.73 m^2^ when using eGFR instead of mGFR, and did not increase with decreasing eGFR (Figure 2B).

**Table 2:** Urinary creatinine excretion rate according to mGFR level, by gender, age, and ethnicity.

Ethnic origin, age, mGFR (mL/min per1.73m^2^)	Men (n = 1072)											Women (n = 537)										
		Urinary creatinine excretion rate											Urinary creatinine excretion rate									
		mmol/kg/24h					mmol/24h						mmol/kg/24h					mmol/24h				
	N	m± sd	10th	25th	75th	90th	m± sd	10th	25th	75th	90th	N	m± sd	10th	25th	75th	90th	m± sd	10th	25th	75th	90th
***Total***							*n = 1072*											*n = 537*				
≥ 60	**188**	0.20±0.03	0.15	0.17	0.22	0.24	15.3±3.1	11.4	13.0	17.0	19.4	**94**	0.16±0.03	0.12	0.14	0.17	0.19	9.6±1.9	7.2	8.3	10.8	12.2
45**–**60	**233**	0.18±0.03	0.14	0.15	0.20	0.22	13.9±3.0	10.6	11.8	15.5	17.9	**88**	0.15±0.03	0.11	0.13	0.16	0.18	9.2±2.1	6.8	7.7	10.6	11.7
30**–**44	**321**	0.17±0.03	0.13	0.14	0.19	0.21	13.2±3.2	9.3	11.0	15.2	17.3	**151**	0.15±0.03	0.10	0.12	0.17	0.19	9.3±2.2	6.8	7.5	10.6	12.3
15**–**29	**274**	0.17±0.04	0.12	0.14	0.19	0.22	12.7±3.2	9.0	10.5	14.6	17.0	**161**	0.13±0.03	0.09	0.11	0.15	0.18	8.8±2.1	6.1	7.3	10.6	11.5
<15	**56**	0.15±0.04	0.11	0.13	0.18	0.20	12.1±3.3	7.7	9.2	14.0	16.8	**43**	0.12±0.03	0.08	0.09	0.14	0.15	7.6±2.5	5.0	5.5	8.9	10.8
***Non-African origin***																						
***<60 years***							*n = 377*											*n = 219*				
≥ 60	**92**	0.20±0.03	0.16	0.18	0.22	0.24	15.3±2.9	11.7	13.0	16.9	19.1	**45**	0.16±0.03	0.12	0.14	0.19	0.22	9.7±1.8	7.6	8.9	10.8	11.9
45**–**60	**78**	0.18±0.03	0.15	0.17	0.20	0.22	14.5±3.0	11.4	12.5	16.0	18.5	**35**	0.16±0.03	0.13	0.13	0.18	0.20	9.4±2.3	6.8	7.8	10.8	11.9
30**–**44	**96**	0.19±0.03	0.15	0.17	0.21	0.22	14.6±3.4	11.0	12.3	16.8	18.6	**61**	0.16±0.04	0.12	0.13	0.18	0.22	9.7±2.4	7.0	8.1	11.0	12.8
15**–**29	**91**	0.17±0.03	0.13	0.15	0.19	0.22	13.2±2.6	10.3	11.4	14.8	16.7	**61**	0.14±0.03	0.10	0.11	0.16	0.19	8.9±2.3	6.0	7.4	10.9	12.0
<15	**20**	0.17±0.03	0.13	0.14	0.20	0.21	13.8±3.0	9.2	12.4	15.1	17.5	**17**	0.13±0.03	0.09	0.11	0.13	0.15	7.9±2.5	5.3	6.1	8.9	11.6
***≥60 years***							*n = 524*											*n = 215*				
≥60	**50**	0.18±0.03	0.14	0.15	0.20	0.21	14.3±2.7	10.7	12.5	15.8	17.4	**27**	0.15±0.02	0.13	0.14	0.15	0.18	8.6±1.4	6.4	7.7	9.5	10.3
45**–**60	**113**	0.17±0.03	0.13	0.15	0.18	0.20	12.9±2.7	10.1	11.2	14.4	16.6	**41**	0.14±0.02	0.11	0.12	0.15	0.16	8.5±1.7	6.7	7.1	9.6	10.6
30**–**44	**186**	0.16±0.03	0.12	0.14	0.18	0.20	12.2±2.6	8.9	10.3	13.9	15.4	**60**	0.13±0.03	0.09	0.11	0.15	0.16	8.3±1.7	6.4	7.0	9.2	10.5
15**–**29	**147**	0.15±0.04	0.11	0.13	0.18	0.20	11.9±3.2	8.3	9.8	13.8	15.7	**73**	0.13±0.03	0.09	0.10	0.14	0.16	8.6±1.8	6.2	7.3	9.8	11.0
<15	**28**	0.14±0.03	0.11	0.12	0.15	0.18	10.9±2.6	7.5	8.5	13.4	14.4	**14**	0.10±0.03	0.07	0.08	0.14	0.15	6.7±2.3	4.0	4.8	8.4	10.0
***African origin***																						
***<60 years***							*n = 112*											*n = 43*				
≥60	**35**	0.21±0.08	0.18	0.19	0.24	0.25	17.4±3.1	13.0	15.7	19.7	20.8	**11**	0.15±0.02	0.12	0.13	0.17	0.17	11.7±1.6	9.8	10.6	12.9	13.1
45**–**60	**28**	0.21±0.04	0.16	0.19	0.23	0.27	16.1±3.2	11.8	14.1	18.3	20.0	**3**	0.17±0.01	0.16	0.16	0.18	0.18	11.6±1.1	10.8	10.8	12.9	12.9
30**–**44	**23**	0.20±0.03	0.16	0.18	0.22	0.24	15.6±3.8	10.5	12.9	18.6	19.8	**14**	0.16±0.03	0.13	0.14	0.18	0.20	10.5±1.7	8.0	9.1	11.7	13.3
15**–**29	**23**	0.22±0.04	0.16	0.19	0.24	0.27	16.4±3.2	12.4	13.2	18.9	19.9	**12**	0.16±0.04	0.10	0.12	0.19	0.20	10.6±1.6	9.8	10.2	11.4	11.5
<15	**3**	0.18±0.03	0.09	0.09	0.24	0.24	14.1±8.0	5.1	5.1	20.0	20.0	**3**	0.12±0.02	0.11	0.11	0.14	0.14	10.1±3.8	7.2	7.2	14.4	14.4
***≥60 years***							*n = 37*											*n = 17*				
≥60	**6**	0.19±0.02	0.16	0.16	0.20	0.22	14.3±0.42	10.8	11.5	17.9	18.6	**1**	-	-	-	-	-	-	-	-	-	-
45**–**60	**11**	0.17±0.04	0.15	0.15	0.19	0.21	13.4±1.7	10.4	11.0	14.8	16.6	**6**	0.14±0.02	0.12	0.12	0.15	0.17	10.0±1.3	8.4	9.1	11.4	11.5
30**–**44	**13**	0.16±0.03	0.13	0.14	0.17	0.20	12.9±1.8	11.7	11.8	13.8	15.2	**6**	0.14±0.03	0.10	0.13	0.17	0.20	10.1±3.6	7.4	7.6	10.3	17.0
15**–**29	**5**	0.16±0.01	0.15	0.16	0.16	0.17	10.9±2.6	8.8	9.8	12.2	13.1	**4**	0.16±0.04	0.11	0.13	0.19	0.20	10.3±2.3	7.8	8.4	12.3	12.5
<15	**2**	0.14±0.02	0.13	0.13	0.15	0.15	10.8±3.4	10.6	10.6	11.2	11.2	**0**	**-**	**-**	**-**	**-**	**-**	**-**	**-**	**-**	**-**	**-**

Abbreviations: mGFR, measured glomerular filtration rate.

### Factors associated with low urinary creatinine excretion rate at baseline

Odds ratios of low urinary creatinine excretion significantly and gradually increased as BMI decreased, both before and after adjusting for demographic variables and mGFR (Table 3). Urinary creatinine excretion was not significantly associated with diabetes and metabolic acidosis. Lower prealbumin and urinary urea levels were significantly associated with higher ORs of low urinary creatinine excretion. In contrast, there was no significant association with serum albumin. Triglycerides were inversely and significantly associated with urinary creatinine excretion. Inflammation, reflected by either higher WBC, plasma fibrinogen or haptoglobin levels, was also inversely and significantly associated with low urinary creatinine excretion after adjusting for confounders, but no such association was observed with CRP and orosomucoid. The higher the quartile of 24-hour proteinuria, the lower the ORs of low urinary creatinine excretion rate.

**Table 3:** Factors associated with low urinary creatinine excretion rate at baseline, n = 1609.

	Crude OR (95% CI)	Age, gender, ethnicity, centre and mGFR-adjusted OR (95% CI)
**BMI group (kg/m^2^)**		
*<18.5*	4.0 (2.1-7.8)	7.5 (3.5–16.3)
*18.5*–*25*	1	1
*25*–*30*	0.53 (0.41–0.69)	0.29 (0.21–0.39)
*30*–*35*	0.45 (0.31–0.65)	0.22 (0.15–0.34)
*≥35*	0.20 (0.09–0.44)	0.11 (0.05–0.26)
Diabetes^a^ (yes *vs* no)	1.3 (0.98–1.6)	0.84 (0.65–1.1)
Metabolic acidosis^b^ (yes *vs* no)	1.3 (0.96–1.8)	0.91 (0.63–1.3)
**Nutritional markers**		
HDL cholesterol <*vs* ≥ 1.3/1.0 mmol/L*	0.73 (0.57–0.93)	0.62 (0.48–0.81)
Triglycerides *≥ vs <*1.7 mmol/L	0.91 (0.71–1.2)	0.68 (0.52–0.89)
Serum albumin <*vs* ≥ 35 g/L	1.1 (0.79–1.5)	0.90 (0.62–1.3)
Prealbumin <*vs* ≥ 0.30 g/L	1.3 (1.03–1.7)	1.5 (1.1–1.9)
Urinary urea by quartiles (mmol/24h)		
*lower*	14.0 (9.2–21.2)	16.4 (10.5–25.7)
*2nd*	4.4 (2.9–6.8)	5.0 (3.2–7.9)
*3nd*	1.9 (1.2–3.0)	2.2 (1.4–3.5)
*upper*	1	1
**Inflammation markers**		
WBC count ≥ *vs* <7.5 10^3^/mm^3^	0.97 (0.75–1.3)	0.74 (0.56–0.98)
Plasma fibrinogen ≥ *vs* <5 g/L	1.1 (0.80–1.6)	0.68 (0.47–0.99)
Haptoglobin ≥ *vs* <90th percentile	0.91 (0.60–1.4)	0.60 (0.38–0.95)
Orosomucoid ≥ *vs* <90th percentile	1.6 (1.0–2.4)	1.1 (0.72–1.8)
CRP ≥ *vs* <10 mg/L	1.3 (0.89–1.9)	0.96 (0.63–1.4)
**Proteinuria by quartiles (g/24h)**		
*lower*	1	1
*2nd*	0.65 (0.48–0.89)	0.50 (0.35–0.71)
*3nd*	0.74 (0.54–1.0)	0.46 (0.32–0.66)
*upper*	0.56 (0.40–0.77)	0.29 (0.19–0.43)

Abbreviations: OR (95% CI), odds-ratios and 95% confidence interval; mGFR, measured glomerular filtration rate; BMI, body mass index; HDL, high-density lipoprotein; WBC, white blood cells; CRP, C-reactive protein.

Cut-off points of urinary urea were: <312.8 mmol/24h, 312.8–382.5 mmol/24h, 382.5–456.3 mmol/24h,>456.3 mmol/24 in men; <252.0 mmol/24h, 252.0–302.1 mmol/24h, 302.1–375.0 mmol/24h,>375.0 mmol/24h in women. Cut-off points of proteinuria urea were: <0.14 g/24h, 0.14–0.35 g/24h, 0.35–1.1 g/24,>1.1 g/24h in men; <0.12 g/24g, 0.12–0.21 g/24h, 0.21–0.89 g/24h,>0.89 g/24h in women.

*Gender-specific thresholds were used to define high-density lipoprotein cholesterol cut-off points: 1.0 mmol/L in men and 1.3 mmol/L in women.

a Fasting glucose ≥ 7 mmol/L or HbA1c ≥6.5 or antidiabetic treatment or reported diabetes.

b Venous CO2 <22 mmol/L or alkaline treatment.

Fully adjusted ORs for low urinary creatinine excretion increased significantly as mGFR decreased (Table 4), but did not with eGFR: ORs were 0.95 (0.59–1.5), 0.90 (0.57–1.4), 0.94 (0.57–1.5), and 0.62 (0.29–1.3) for eGFR of 45–60, 30–44, 15–29, and <15 as compared with ≥60 mL/min/1.73 m^2^. Other factors significantly associated with low creatinine excretion in the multivariate model were age, gender, ethnicity, BMI, 24-hour urinary urea and proteinuria. While the association with metabolic acidosis remained nonsignificant (data not shown), it is noteworthy that diabetes became strongly associated with low urinary creatinine excretion after adjustment for the other risk factors. Similar associations were observed in patients with and without diabetes except for pre-albumin, which was related to low urinary creatinine excretion only in those with diabetes.

**Table 4:** Multivariate analysis of factors associated with low urinary creatinine excretion rate at baseline, by diabetes status.

OR (95% CI)			
	Overall population	Without diabetes	With diabetes
	(n = 1609)	(n = 1153)	(n = 456)
**Age, year**	1.05 (1.04–1.06)	1.05 (1.04–1.07)	1.05 (1.02–1.07)
**Gender (men vs women)**	2.1 (1.5–2.9)	1.9 (1.3–2.9)	2.1 (1.1–3.9)
**African vs non-African origin**	0.28 (0.16–0.49)	0.42 (0.22–0.80)	0.15 (0.05–0.44)
**mGFR (ml/min per1.73m2)**			
* ≥60*	1	1	1
* 45*–*60*	2.4 (1.3–4.2)	2.1 (1.1–4.2)	2.5 (0.79–7.9)
* 30*–*44*	3.6 (2.1–6.2)	3.2 (1.7–6.2)	3.8 (1.3–11.0)
* 15*–*29*	5.2 (2.9–9.2)	5.0 (2.5–10.0)	4.7 (1.6–14.5)
* <15*	9.2 (4.3–19.6)	8.2 (3.3–20.5)	11.9 (2.6–55.4)
**Diabetes mellitus**			
* no*	1	-	-
* yes*	1.8 (1.3-2.6)	-	-
**BMI group (kg/m^2^)**			
*<18.5*	9.6 (4.2–21.9)	9.7 (4.1–22.9)	
*18.5–25*	1	1	5.0 (2.4–10.4)
*25–30*	0.29 (0.20–0.41)	0.32 (0.21–0.48)	1
*30–35*	0.24 (0.15–0.39)	0.12 (0.06–0.26)	1.6 (0.81–3.0)
*≥35*	0.12 (0.05–0.32)	0.12 (0.03–0.53)	0.51 (0.15–1.8)
**Prealbumin *(g/L)***			
* >0.30*	1	1	1
* <0.30*	1.2 (0.84–1.7)	0.83 (0.54–1.3)	2.3 (1.3–4.2)
**Urinary urea (mmol/24h)**			
*lowest*	13.6 (8.3–22.1)	17.8 (9.0–35.5)	10.6 (4.8–23.4)
*2nd*	4.2 (2.6–6.9)	5.1 (2.5–10.2)	3.7 (1.7–7.8)
*3rd*	2.0 (1.2–3.4)	2.5 (1.2–5.2)	1.6 (0.75–3.5)
*highest*	1	1	1
**Proteinuria (g/24h)**			
*lowest*	1	1	1
*2nd*	0.63 (0.42–0.94)	0.65 (0.40–1.0)	0.58 (0.25–1.4)
*3rd*	0.52 (0.34–0.81)	0.51 (0.30–0.86)	0.63 (0.27–1.4)
*highest*	0.37 (0.23–0.60)	0.38 (0.21–0.69)	0.37 (0.16–0.86)

Abbreviations: OR (95% CI), odds-ratios and 95% confidence interval; mGFR, measured glomerular filtration rate; BMI, body mass index.

Cut-off points of urinary urea were: <312.8 mmol/24h, 312.8–382.5 mmol/24h, 382.5–456.3 mmol/24h,>456.3 mmol/24 in men; <252.0 mmol/24h, 252.0–302.1 mmol/24h, 302.1–375.0 mmol/24h,>375.0 mmol/24h in women. Cut-off points of proteinuria urea were: <0.14 g/24h, 0.14–0.35 g/24h, 0.35–1.1 g/24,>1.1 g/24h in men; <0.12 g/24g, 0.12–0.21 g/24h, 0.21–0.89 g/24h,>0.89 g/24h in women.

*Multivariate adjustment, including center.

**Table 5:** Linear mixed model analysis of factors associated with mean differences in the level (in mmol/24h) and change over time (in mmol/24h per year) in urinary creatinine excretion rate.

Mean difference in urinary creatinine excretion (95% Confidence Interval)	
Intercept, mmol/24h *	12.68(12.39,12.98)
Factors associated with baseline urinary creatinine	
Age, year	−0.06(−0.07,−0.05)
Women vs men	−3.57(−3.83,−3.32)
African vs non African origin	1.87(1.57,2.18)
mGFR, per each 10 ml/min/1.73 m^2^ decrease	−0.23(−0.29,−0.17)
Diabetes	−0.38(−0.63,−0.13)
BMI, kg/m^2^	
<18.5	−1.54(−2.21,−0.88)
18.5–25	0 (ref)
25–30	1.11(0.87,1.34)
30–35	1.71(1.39,2.02)
>35	1.91(1.41,2.42)
Urinary urea, per each 10 mmol/day decrease	−0.09(−0.10,−0.08)
Proteinuria	
lowest	0 (ref)
2^nd^	0.42(0.13,0.71)
3^rd^	0.66(0.36,0.96)
highest	0.80(0.48,1.12)
	
Slope, mmol/24h per year**	−0.19(−0.24,−0.14)
Factors associated with changes in urinary creatinine	
Women vs Men	0.16(0.08,0.25)
mGFR, per each 5 ml/min/1.73m^2^ per yr decrease^†^	−0.29(−0.34,−0.25)
Urinary urea, per each 25 mmol/24h per yr decrease^†^	−0.15(−0.17,−0.14)

*mean predicted level in urinary creatinine excretion rate in mmol/24h for 60-yr old non-African and non-diabetic men, with normal BMI (within 18.5 to 25 kg/m^2^), baseline mGFR of 40 ml/min/1.73 m^2^, urinary urea of 400 mmol/d and proteinuria in the lowest quartiles.

** mean predicted change in urinary creatinine excretion rate in mmol/24h per year in men with stable mGFR and urinary urea, i.e., with no change in level from baseline.

† 5 ml/min/1.73 m^2^ per yr decrease in mGFR and 25 mmol/24h per yr decrease in urinary urea correspond to about three times the mean annual decrease for each variable.

### Factors associated with baseline rate and annual change in urinary creatinine excretion

As expected, factors associated with lower mean creatinine excretion rate at baseline were similar to those found above with the logistic model. Each 10 mL/min/1.73 m^2^ decrease in mGFR was associated with 0.23±0.03 mmol/24 h decrease in urinary creatinine excretion rate, and each 10 mmol/24h decrease in urinary urea with 0.09±0.00 mmol/24 h (Table 5). Patients with proteinuria in the highest quartile had a higher rate of 0.80±0.16 mmol/24 h than those in the lowest quartile.

Mean annual decline in mGFR was 1.53±0.12 mL/min/1.73 m^2^ and mean annual decrease in urinary urea was 7.42±1.22 mmol/24 h. Mean change in urinary creatinine excretion rate was 0.28±0.02 mmol/24 h per year and did not differ according to baseline characteristics except gender: women had slower mean annual decrease than men. Patients with a fast annual decline in mGFR of 5 mL/min/1.73 m^2^ (about 3 times the mean level) had a decrease in urinary creatinine excretion more than twice as big than those with stable mGFR, independent of changes in protein intake assessed by urinary urea as well as of other determinants.

## Discussion

This study showed that decrease in urinary creatinine excretion may occur early in CKD independent of nutritional factors and protein intake. As expected, the study confirmed the impact of age, ethnicity and nutritional factors on urinary creatinine excretion, but also found a graded relationship with proteinuria and no significant association with inflammation. Together with the range of urinary creatinine excretion provided by age, gender, ethnicity as well as by mGFR, these findings have important clinical implications regarding management of muscle mass loss and validation of urine collection in CKD patients.

Very few studies have investigated the relation between muscle loss and kidney function decline. Foley *et al*. [28] reported a high prevalence (27.2%) of reduced muscle mass assessed by bioimpedance in community-dwelling adults. Although its prevalence increased as estimated glomerular filtration rate (eGFR) decreased, the association was no longer significant after adjustment for age and comorbidity. Nevertheless, these results are difficult to compare with ours, because bioimpedance measures not only muscle mass but also body water [38], [45]. In the Modification of Diet in Renal Disease (MDRD) study [30], several nutritional parameters, including urinary creatinine, showed lower values for patients with mGFR <21 ml/min/1.73 m^2^ than for those with mGFR>37 ml/min/1.73 m^2^. The associations, however, were strongly attenuated after controlling for protein and energy intakes leading the authors to conclude that they were mediated by the lower dietary intake associated with kidney function decline. In contrast, *Micco et al* [17] showed an inverse cross-sectional association between urinary creatinine excretion and CKD stages from 3 to 5 independent of nutritional factors as well as a decrease over time in urinary creatinine excretion independent of eGFR decline. The NephroTest study extends these findings to earlier CKD stages, showing that low creatinine excretion rate was indeed twice as common in stage 3a (GFR within 45–60 mL/min/1.73 m^2^) than in stages 1–2 (GFR ≥60 mL/min/1.73 m^2^), independent of both nutritional parameters and protein intake assessed by urinary urea. Although an increase in extrarenal excretion of creatinine, including its conversion to other metabolites and its breakdown by gut bacteria [39], [43] cannot be ruled out as an explanation of the above association, this is unlikely because it is usually significant in the latest stage of CKD. Similarly, while it is well-known that the relative participation of tubular secretion in creatinine excretion increases with kidney function decline, this has definitely no impact on the overall urinary creatinine excretion in steady state such as that of patients with CKD.

As expected and in line with other studies[13], [18], [26]–[28], [30], [39] age, gender and ethnicity were major determinants of urinary creatinine excretion rate. Moreover, the large sample size of the NephroTest study enabled for the first time to document the range of urinary creatinine excretion rate not only according to these variables as previously reported, but also according to mGFR level. These values could thus be used by clinicians to validate 24-hour urine collection in patients with reduced kidney function.

A large set of nutritional measures were available in this study. BMI has been reported to be significantly associated with muscle mass in both transplanted [20] and dialysis patients [14], [19]. In line with other studies of CKD [13], [17], [18], [25] and non CKD populations [26], [27], we observed that urinary creatinine excretion was significantly lower with lower BMI. Prealbumin is also considered as an important nutritional marker [1], [2], [6], [9], [14], [37], [46]. We found that lower urinary creatinine levels were significantly associated with a low prealbumin level in patients with diabetes, but not in those without. The number of missing data for prealbumin, however, may have weakened the latter association. In contrast, serum albumin was not associated with urinary creatinine excretion. Although it has been shown to be positively associated with total muscle mass estimated from X-ray absorptiometry [29], its usefulness as a nutritional marker has been challenged in end-stage renal disease (ESRD) patients[3], [47], [48]. Similarly, we failed to find that urinary creatinine excretion was significantly associated with transferrin, homocysteine, total and HDL cholesterol or triglycerides. As expected, urinary urea was significantly and positively associated with creatininuria in our study. Indeed, protein intake is an important determinant of muscle mass [28], [39]. Our finding that the decrease in urinary creatinine excretion over time is associated with mGFR decline independent of both nutritional measurements and lowering protein intake suggests that other mechanisms than reduced dietary intakes may explain muscle wasting in CKD.

Only sparse data are available about the potential impact of diabetes on muscle mass loss. *Cano et al*. [24] reports lower lean body mass estimated from creatinine generation in diabetic *vs* nondiabetic patients on hemodialysis. *Pupim et al*. [21] also showed that the loss of lean body mass accelerates in dialysis patients with diabetes. Mechanisms related to diabetes mellitus [32], [37] have been suggested to explain this relation. *Siew et al*. [33] demonstrated a significant association between skeletal muscle protein breakdown and insulin resistance in nondiabetic chronic hemodialysis patients. In agreement with other studies [18], [27], we found significantly lower urinary creatinine excretion in CKD patients with than without diabetes mellitus. Metabolic acidosis has also been shown to be associated with malnutrition-inflammation in end-stage CKD [35]. Its occurrence in the very late stages of CKD[49] may explain why we found no relation between low muscle mass and acidosis.

Although inflammation frequently accompanies poor nutritional status [1], [7], [14], [31], [36], [48], [50] and plays an important role in the pathophysiology of muscle wasting [36], [50], there are conflicting results about the association of inflammatory status with muscle mass loss [36]. The elevated CRP levels often observed in CKD patients [7], [8], [11], [12] reflect the generation of proinflammatory cytokines, which may contribute to muscle wasting by stimulating protein catabolism. In disagreement with some [14], [19], but not all studies [18], [28], inflammation defined by CRP ≥10 mg/L was not significantly associated with urinary creatinine excretion rate after adjusting for nutritional factors in this study. Importantly, there was no association with either of the other available inflammatory markers, including orosomucoid, haptoglobin, plasma fibrinogen, or WBC after multivariate adjustment. In contrast, there was a graded and independent relationship with 24-hour proteinuria which, to our knowledge, has not been described before. The complex associations of proteinuria with protein wasting and inflammation may explain the observed association. Further studies are needed to elucidate whether muscle wasting is related to inflammation in non-end-stage CKD or whether it depends on the type of malnutrition, as suggested in dialysis patients [48].

The strengths of this study include the use of a gold-standard method to measure renal function (mGFR), the ability to assess urinary creatinine excretion based on both carefully collected urine during a 5-hour visit and 24-hour urine collection as well as the availability of repeated measurements of these markers as well as of several potential determinants of muscle wasting that may have confounded the association of interest. It is worth noting that we found no association of urinary creatinine excretion with GFR estimated from the CKD-EPI equation, which is explained by the impact of creatinine generation on both serum and urinary creatinine resulting in overestimation of eGFR as compared with mGFR in patients with low urinary creatinine, and conversely, underestimation in those with high urinary creatinine. This study also has limitations. First, we had no information about physical activity [22], [28], [31], which might have explained some of our findings. Second, although no direct measure of muscle mass exists, it might be considered a limitation that we used urinary creatinine excretion as a measure of muscle mass instead of reference methods such as computerized tomography (CT) or magnetic resonance imaging (MRI) [40], [41]. However, the time and money required for these methods make them impractical in large-scale clinical studies. Finally, demographic- and anthropometric parameter-based equations [51]–[53] used to estimate muscle mass in individuals with normal kidney function are inappropriate in the CKD population because they do not take into account the decrease in urinary creatinine excretion with reduced kidney function.

## Conclusions

Our findings suggest that decrease in urinary creatinine excretion rate may appear early in CKD patients, independent of decreased protein intake assessed by urinary urea excretion, as well as of other determinants of muscle mass loss including gender, an older age, non-African origin, diabetes, lower BMI and 24-h proteinuria levels. Urinary creatinine concentration is a useful tool, easy to apply routinely. Reference values provided by age, gender, and GFR level might help clinicians to identify individuals who may need both special interventions, including nutritional or exercise advice, and monitoring of their response to these interventions. The decrease of urinary creatinine excretion with GFR decline also questions the accuracy of normalizing for creatininuria when estimating urine analytes concentration which may be overestimated in CKD patients with reduced muscle mass.
